# Poultry and beef meat as potential seedbeds for antimicrobial resistant enterotoxigenic *Bacillus* species: a materializing epidemiological and potential severe health hazard

**DOI:** 10.1038/s41598-018-29932-3

**Published:** 2018-08-02

**Authors:** Kamelia M. Osman, Anthony D. Kappell, Ahmed Orabi, Khalid S. Al-Maary, Ayman S. Mubarak, Turki M. Dawoud, Hassan A. Hemeg, Ihab M. I. Moussa, Ashgan M. Hessain, Hend M. Y. Yousef, Krassimira R. Hristova

**Affiliations:** 1 Department of Microbiology, Faculty of Veterinary Medicine, Cairo University, Giza, Egypt; 2 Department of Civil, Construction, and Environmental Engineering, Marquette University, Milwaukee, WI USA; 3 Department of Botany and Microbiology, College of Science, King Saud University, Riyadh, Saudi Arabia; 4 Department of Clinical Laboratory sciences, college of Applied Medical sciences, Taibah University, Taibah, Saudi Arabia; 5 Department of Health Science, College of Applied Studies and Community Service, King Saud University, Riyadh, Saudi Arabia; 6 Central Administration of Preventive Medicine, General Organization for Veterinary Service, Giza, Egypt; 7 Department of Biological Sciences, Marquette University, Milwaukee, WI USA

## Abstract

Although *Bacillus cereus* is of particular concern in food safety and public health, the role of other *Bacillus* species was overlooked. Therefore, we investigated the presence of eight enterotoxigenic genes, a hemolytic gene and phenotypic antibiotic resistance profiles of *Bacillus* species in retail meat samples. From 255 samples, 124 *Bacillus* isolates were recovered, 27 belonged to *B*. *cereus* and 97 were non-*B*. *cereus* species. Interestingly, the non-*B*. *cereus* isolates carried the virulence genes and exhibited phenotypic virulence characteristics as the *B*. *cereus*. However, correlation matrix analysis revealed the *B*. *cereus* group positively correlates with the presence of the genes *hblA*, *hblC*, and *plc*, and the detection of hemolysis (*p* < 0.05), while the other *Bacillus* sp. groups are negatively correlated. Tests for antimicrobial resistance against ten antibiotics revealed extensive drug and multi-drug resistant isolates. Statistical analyses didn’t support a correlation of antibiotic resistance to tested virulence factors suggesting independence of these phenotypic markers and virulence genes. Of special interest was the isolation of *Paenibacillus alvei* and *Geobacillus stearothermophilus* from the imported meat samples being the first recorded. The isolation of non-*B*. *cereus* species carrying enterotoxigenic genes in meat within Egypt, suggests their impact on food safety and public health and should therefore not be minimised, posing an area that requires further research.

## Introduction

The diverse genus *Bacillus* includes harmless environmental and pathogenic species. The *B*. *cereus* group are closely related including *B*. *anthracis*, *B*. *thuringiensis*, *B*. *mycoides*, and *B*. *cereus* which are known pathogens or opportunistic pathogens to humans^1^. The *B*. *subtilis* group, including *B*. *mojavensis*, *B*. *pumilus*, *B*. *fusiformis*, *B*. *licheniformis* and *B*. *subtilis*, are common to soil^1^. The *B*. *cereus* and *B*. *subtilis* groups have been implicated in food poisoning as a result of intoxication^1^, either by the consumption of food containing pre-formed toxin or toxins produced by these bacteria in the human gut^2–5^. *Bacillus circulans*, *B*. *lentus*, *B*. *amyloliquefaciens*, *B*. *simplex*, *B*. *firmus*, and *B*. *megaterium*, have been rated as insignificant and disregarded in food poisoning episodes, however their presence and consequent production of enterotoxins and emetic toxins has been increasingly documented and confirmed by cellular assays^6^. Other aerobic spore-forming bacteria including the genera *Sporosarcina*, *Paenisporosarcina*, *Brevibacillus*, *Paenibacillus*, and *Geobacillus* have the potentiality to form biofilms within pipes and stainless steel equipment and to the resist industrial pasteurization posing a hazard for the food industry.

The presence of environmental isolates of *Bacillus* spp. harboring one or more enterotoxin gene holds crucial importance for the food safety, however, evaluation of toxin gene presence and toxin activity in *Bacillus* spp. other than *B*. *cereus* has not been thoroughly investigated. The diarrheal enterotoxins, Cytotoxin K (encoded by the *cytK* gene) and enterotoxin FM (encoded by the *entFM* genes), hemolysin BL (encoded by the *hbl* operon) and non-hemolytic enterotoxin (encoded by the *nhe* genes) are regarded as the main enterotoxins involved in foodborne illness studied in *B*. *cereus*^7,8^. There is a lack of information on the presence of these enterotoxins in the non-*B*. *cereus* groups.

As Egypt depends on imported beef meat, which reached 200,200 metric tons in the year 2016^9^, to complement the animal protein demands, it is crucial to assess the safety of both imported meat and local products. There is increasing concern about imported meat and question whether Egypt’s food safety system can protect them from tainted foreign products. Compared to the U.S.A., EU or Australia, the food safety standards in Egypt and other underdeveloped countries are not as high. The aim of this study was to determine if the presence of enterotoxins and other putative virulence factors are contributing to the total cytotoxicity of the enterotoxic *Bacillus* spp. isolated from retail meat in the Egyptian market. Specifically, we have determined incidence and level of contamination with *B*. *cereus* and non-*B*. *cereus* groups in retail chicken and local and imported beef. We performed a comparative overview on the observed hemolysin BL production, cytotoxic activity, swarming, ability to form biofilms, phenotypic antibiotic resistance profile and enterotoxin gene profile (NHE, HBL and CytK)^10–12^.

There is an intricate link between metabolism, swarming, biofilm production, antibiotic resistance and virulence. Two soluble virulence factors, lecithinase and amylase production, were noted as phenotypic virulence markers^13,14^. Swarming motility was tested because it is generally considered a precursor step to biofilm formation and a major factor in pathogenesis by many human pathogens^15^. The presence of swarming generally increases virulence as movement over surfaces enable bacteria to migrate from sites of infection, offers protection from macrophages as swarming cells were shown to have enhanced resistance to engulfment, and become resistant to a broad range of antibiotics during swarming^16–18^. Biofilms are considered to promote adhesion and protect cells from antimicrobials and other external insults^19^. Amyloid-like fibrils have been shown to increase the biofilms’ structural integrity and allow staining with certain dyes such as Congo red^20–24^. However, results depending on phenotypic markers alone lack reliability in determining the virulence of *Bacillus* strains.

In this study, we carried out a *m*-PCR to detect the genes for three pore-forming enterotoxins, responsible for the diarrheal type of food poisoning: hemolysin BL (HBL), non-hemolytic enterotoxin (NHE), cytotoxin K (CytK) and enterotoxin FM (EntFM), and the hemolytic gene encoding phospholipase (*plc*)^1,10–12^ in *B*. *cereus* (n = 24) and non-*B*. *cereus* isolates (n = 97) collected from retail chicken local and imported beef meat. In addition, we calculated the level of antibiotic resistance and the multiple-antibiotic resistance indices of isolates present in meat samples.

## Results

### Prevalence

**Figure 1:**
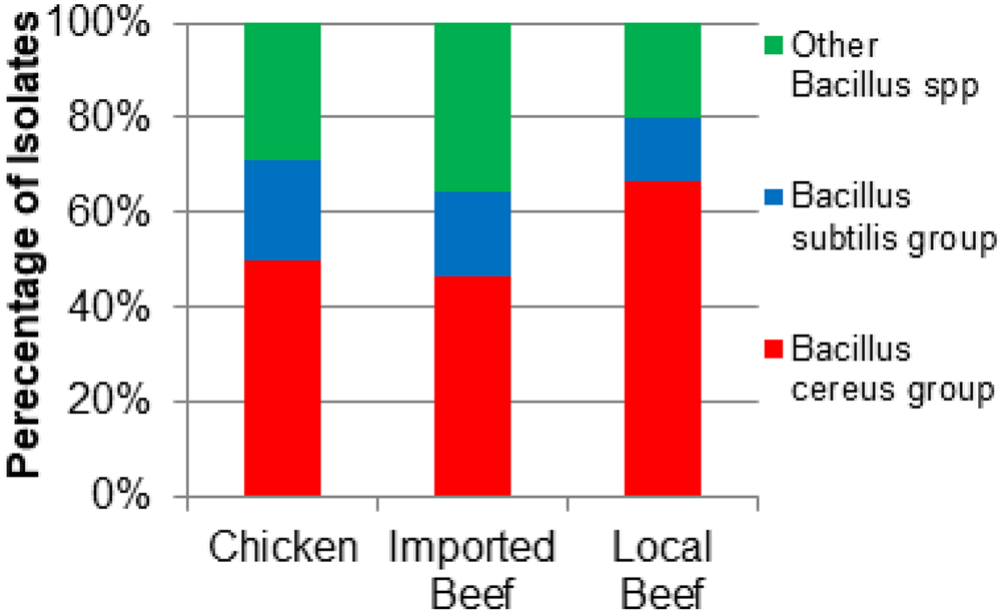
Distribution of isolates from the different sources of meat within the different *Bacillus* spp. groups. (Chicken, n = 66; Imported Beef, n = 28, Local Beef, n = 30).

**Figure 2:**
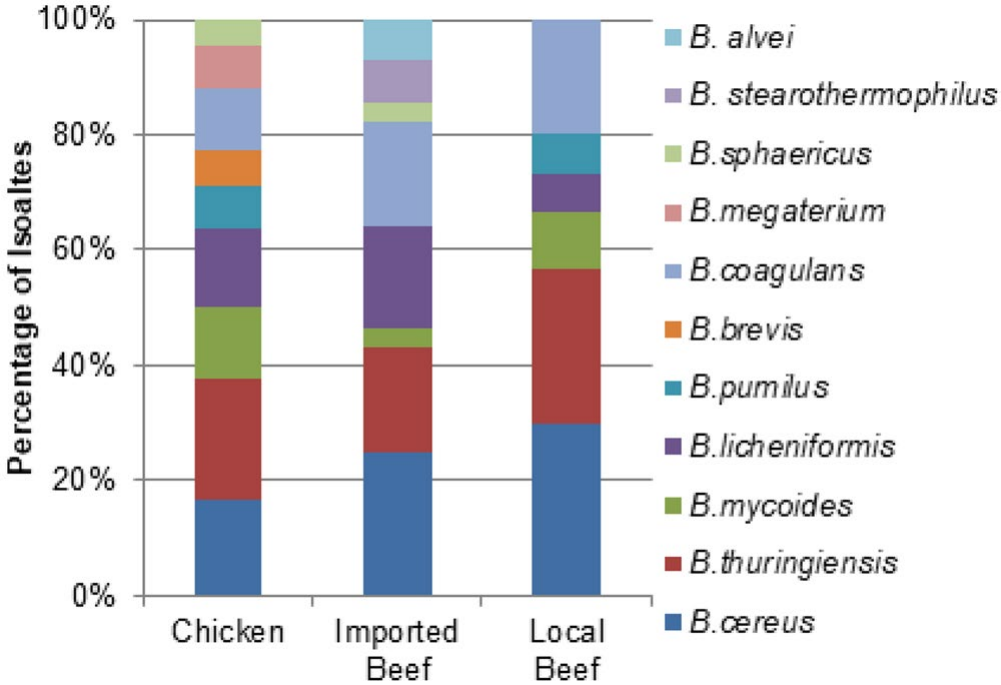
Distribution of isolates of different *Bacillus* species isolated from the different sources of meat. (Chicken, n = 66; Imported Beef, n = 28; Local Beef, n = 30).

The 255 meat samples yielded 124 *Bacillus* spp. isolates containing 66 *B*. *cereus* group isolates (53.2%; *B*. *cereus*, *B*. *thuringiensis*, and *B*. *mycoides*,), 23 *B*. *subtilis* group isolates (18.5%; *B*. *licheniformis* and *B*. *pumilus*) and 35 other *Bacillus* spp. (28.2%; *B*. *coagulans*, *B*. *megaterium*, *B*. *sphaericus*, *B*. *brevis*, *G*. *stearothermophilus*, and *P*. *alvei*) (Fig. 1). The prevalence of the *B*. *cereus* group was 50% (33/66) of isolates within retail chicken, 66.7% (20/30) of isolates from local beef, and 46.4% (13/28) of imported meat isolates (Fig. 1). The *B*. *subtilis* group represented 21.2% (14/66), 13.3% (4/30), and 17.9% (5/28), of isolates from retail chicken, local, and imported beef, respectively. Other *Bacillus* spp. were isolated from chicken, local beef, and imported beef as 28.8% (19/66), 20.0% (6/30), and 35.7% (10/28) of the isolates, respectively. Out of the 124 *Bacillus* isolates, 66 isolates were obtained from 156 chicken meats samples and 58 isolates were recovered from the 99 beef meat. There was no significant difference between source of isolates (chicken, local and imported beef meat) in relation to the different species isolated (*p* > 0.06), except for *G*. *stearothermophilus* and *P*. *alvei* which were signifcantly greater in imported beef meat compared to chicken meat (*p* = 0.014) (Fig. 2).

### Phenotypic assessment of virulence factors

Biofilm production was observed in 105/124 isolates (84.5%) (Table 1). Among the biofilm producers, the abundance was 89.4% in the chicken isolates, 83.3% in the local beef meat, and 75% in the isolates from imported frozen meat.

**Table 1:** Phenotypes virulence factors distribution throughout the *B. cereus* and non-*B. cereus* strains isolated from chicken and beef meat samples.

Source of samples	*Bacillus* groups	*Bacillus* species	Virulence phenotypes							
			Congo red agar test	Biofilm production			Hemolysis			Cytotoxicity
				Weak	Moderate	Strong	α-	β-	γ-	
**Chicken (n = 66)**										
	*Bacillus cereus* group	*cereus* (n = 11)	7	2	3	4	—	11	—	10
		*thuringiensis* (n = 14)	10	3	7	2	—	14	—	10
		*mycoides* (n = 8)	6	1	4	3	1	7	—	7
	*Bacillus subtilis* group	*licheniformis* (n = 9)	6	3	3	2	1	8	—	4
		*pumilus* (n = 5)	2	2	—	2	—	5	—	2
	Other *Bacillus* spp	*coagulans* (n = 7)	4	3	3	1	3	4	—	4
		*megaterium* (n = 5)	3	2	2	1	1	4	—	3
		*sphaericus* (n = 3)	3	—	1	2	—	—	3	3
	*Bervibacillus*	*brevis* (n = 4)	3	1	2	—	—	4	—	3
		**Total**	42 (63.6%)	17 (25.6%)	25 (37.9%)	17 (25.6%)	6 (9.1%)	57 (86.4%)	3 (4.6%)	46 (69.7%)
**Local beef meat (n** = **30)**										
	*Bacillus cereus* group	*cereus* (n = 9)	6	2	4	3	1	8	—	9
		*thuringiensis* (n = 8)	5	1	2	2	—	8	—	6
		*mycoides* (n = 3)	2	1	2	—	3	—	—	3
	*Bacillus subtilis* group	*licheniformis* (n = 2)	2	—	—	2	—	2	—	2
		*pumilus* (n = 2)	2	—	—	1	1	1	—	1
	Other *Bacillus* spp	*coagulans* (n = 6)	3	2	1	2	1	3	2	4
		**Total**	20 (66.7%)	6 (20%)	9 (30%)	10 (33.3%)	6 (20%)	22 (73.3%)	2 (6.7%)	25 (83.3%)
**Frozen beef meat (n** = **28)**										
	*Bacillus cereus* group	*cereus* (n = 7)	3	4	1	—	1	6	—	6
		*thuringiensis* (n = 5)	2	2	—	1	1	3	1	2
		*mycoides* (n = 1)	—	1	—	—	—	1	—	1
	*Bacillus subtilis* group	*licheniformis* (n = 5)	4	1	3	1	1	4	—	4
	Other *Bacillus* spp	*coagulans* (n = 5)	4	1	1	2	3	1	1	3
		*sphaericus* (n = 1)	1		1	—	—	—	1	1
	*Geobacillus*	*stearothermophilus* (n = 2)	1	1	—	1	1	1	—	2
	*Paenibacillus*	*alvei* (n = 2)	—	—	—	—	1	1	—	2
		**Total**	15 (53.6%)	10 (35.7%)	6 (21.4%)	5 (17.9%)	8 (28.6%)	17 (60.7%)	3 (10.7%)	21 (75%)
**Total (n** = **124)**			77 (62.1%)	33 (26.6%)	40 (32.3%)	32 (25.8%)	20 (16.1%)	96 (77.4%)	8 (6.5%)	92 (74.2%)

The other virulence factors as indicated in Table 1, reveals that 92/124 (74.2%) of the total *Bacillus* species isolated were cytotoxic to the Vero cells. In addition, β-hemolysis activity was observed on sheep blood agar in 77.4% of isolates, α-hemolysis activity in 16.1%, and the γ-hemolysis activity in 6.5% of the isolates.

### Distribution of virulence genes among the *B*. *cereus* and non-*B*.*cereus* isolates

**Figure 3:**
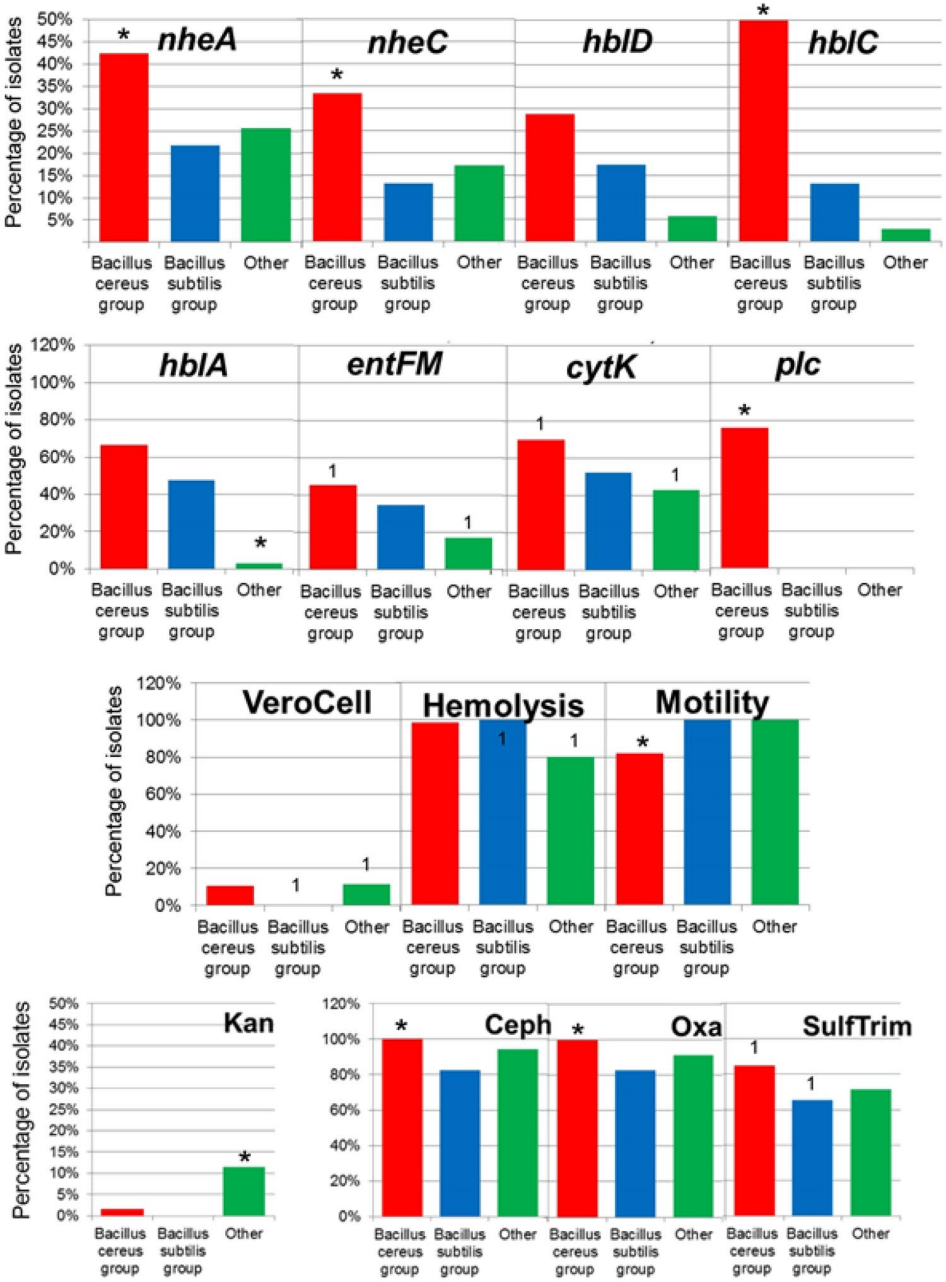
Percentage of isolates showing virulence genes, Vero cell, hemolysis, motility and antibiotic resistance (Kanamycin, KAM; Cephalothin, CEPH; Oxacillin, OXA; Sulfamethazole/Trimethoprim, SulfTrim) between *Bacillus* groups. Asterisks represent a group significantly different from the other two groups (p < 0.05) and numbers (example: “1”) represent groups that are significantly different from each other (p < 0.05). (B. cereus group, n = 66; B. subtilis group, n = 23; Other Bacillus group, n = 35).

Eight virulence genes encoding enterotoxins were targeted in the *Bacillus* isolates based on PCR detection (Table S1). PCR detection included genes encoding hemolytic (*hblA*, *hblC*, and *hblD*) and non-hemolytic (*nheA*, *nheB*, and *nheC*) enterotoxin complexes, cytotoxin K (*cytK*), enterotoxin FM (*entFM*) and one hemolytic gene encoding phospholipase (*plc*) (Fig. 3). At least one of the nine genes were detected in all the isolates except one *B*. *sphaericus* isolated from the frozen imported beef meat. The isolated *Bacillus* commonly possessed *cyt*k (58.9%) as the most prevalent toxin gene followed by *hblA* (45.2%), *plc* (40.3%) and *entFM* (35.5%) genes while the *nheB* gene was the least present (12.9%).

The non-hemolytic genes *nheA* and *nheC*, and the hemolytic enterotoxin HBL complex gene *hblC* were significantly more prevalent in the *B*. *cereus* group isolates compared to the *B*. *subtilis* group, and the other *Bacillus* spp. isolates (*p* < 0.05). There was no significant difference between the number of isolates carrying the *hblA* hemolytic enterotoxin gene in the *B*. *cereus* group compared to *B*. *subtilis* group (*p* = 0.055). The other *Bacillus* spp. isolates showed a significantly lower abundance of the *hblA* gene compared to the *B*. *cereus* and *B*. *subtilis* groups (*p* < 0.05). There was no significant difference in the presence of the *nheB* gene between the groups (*p* > 0.13). In regard to the source of isolated *Bacillus* spp., there were significantly fewer incidences of the enterotoxin FM genes (*entFM*) within the isolates from raw chicken compared to fresh local or imported frozen meat (*p* < 0.05).

There were no significant difference in slime (CR) and biofilm (CV) production of the isolates in the different *Bacillus* spp. groups (p > 0.05). More isolates from the *B*. *cereus* group had a gene encoding phospholipase (*plc*), lecithinase activity, amylase activity, and significantly fewer isolates showing swarming (Fig. 3; *p* < 0.05).

### Antimicrobial resistance prevalence

The isolates were tested for susceptibility against 10 antibiotics representing 8 classes (Table S2). The classes were glycopeptides (vancomycin), β-lactams (penicillin, oxacillin, cephalothin), quinoline (nalidixic acid), sulphonamides (sulfamethoxazole/trimepthoprim), phenicols (chloramphenicol), tetracyclines (tetracycline), aminoglycosides (kanamycin) and macrolids (erythromycin). The 124 isolates displayed a very high level of antibiotic resistances. The highest resistance was displayed to penicillin G (124/124, 100%) while the lowest resistance was recorded against the clinically important antibiotic vancomycin (2/124, 1.6%). A resistance >90% was then recorded to cephalothin and oxacillin. The 124 isolates were resistant to sulfamethazole/trimethoprim, nalidixic acid, erythromycin and tetracycline at 77.4%, 62.9%, 20.2% and 17.74%, respectively. Resistance to kanamycin was lower (4%). Resistance against chloramphenicol, an antibiotic still in use in clinic in Egypt was evidently low, with a total of 3.2% isolates being resistant (3/66 isolates from the chicken and 1/30 isolates from local beef meat).

**Figure 4:**
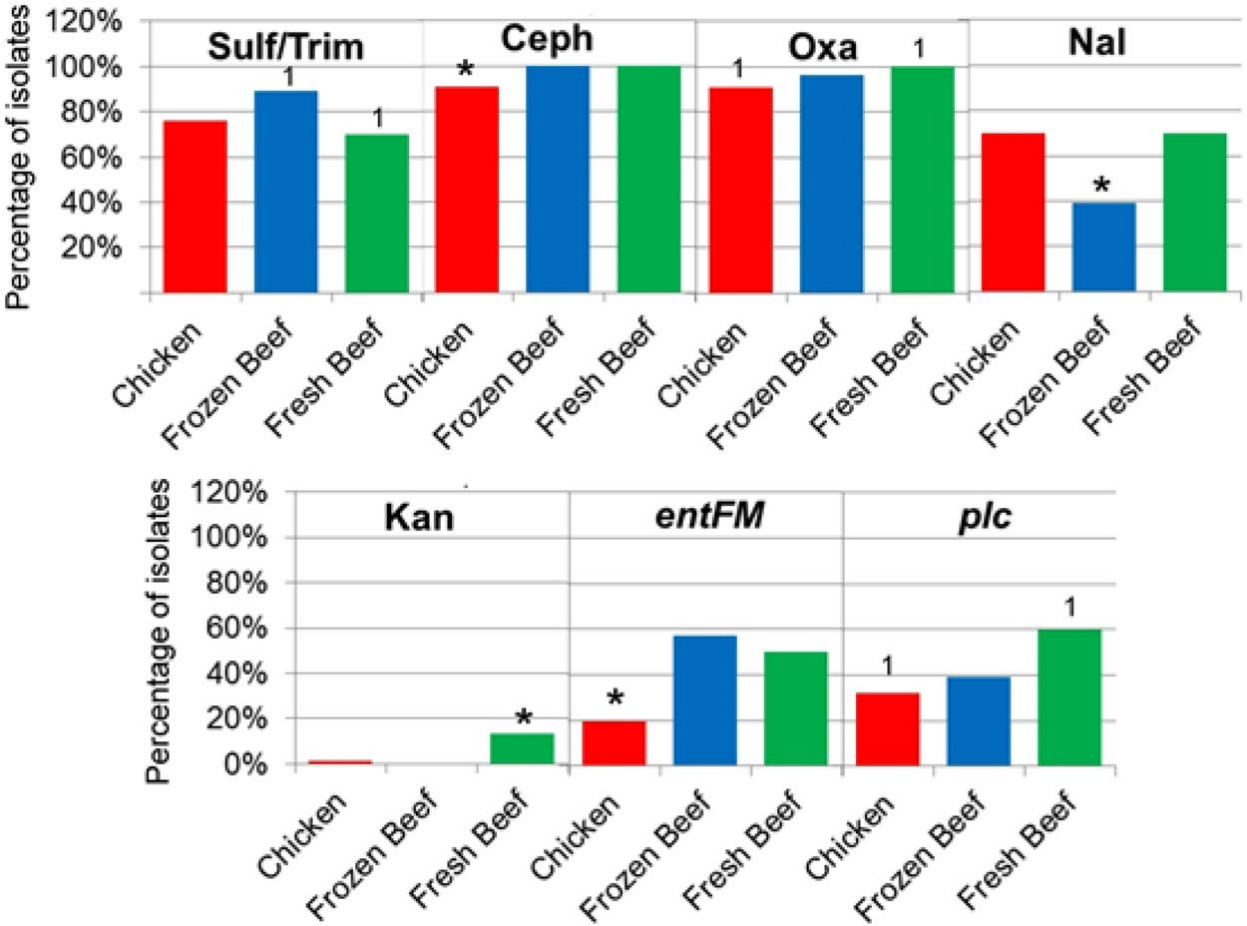
Percentage of isolates within the different meat sources demonstrating antibiotic resistance (Kanamycin, KAM; Cephalothin, CEPH; Oxacillin, OXA; Nalidixic acid, NAL; Sulfamethazole/Trimethoprim, SulfTrim) and virulence genes. Asterisks represent a group significantly different from the other two groups (p < 0.05) and numbers (example: “1”) represent groups that are significantly different from each other (p < 0.05). (Chicken, n = 66; Imported Beef, n = 28; Local Beef, n = 30).

As for the differences between the *Bacillus* groups, prevalence of antibiotic resistance in *P*. *alvei* and *G*. *stearothermophilus* are notably lower compared to other species. Resistance prevalence in *P*. *alvei*, *G*. *stearothermophilus* did not significantly differ compared with *B*. *cereus*, *B*. *thuringiensis*, *B*. *mycoides*, *B*. *licheniformis*, and B. *coagulans*, although this observation may be due to the low number of these species isolated. Resistance to the antibiotics oxacillin, cephalothin, and the antibiotic combination sulfamethazole/trimethoprim was significant more prevalent within isolates of the *Bacillus cereus* group compared to the *B*. *subtilis* group and other *Bacillus* spp. isolates (Fig. 4, *p* < 0.05). There was no significant difference in the percentage of isolates with antibiotic resistance to vancomycin, nalidixic acid, chloramphenicol, tetracycline, and erythromycin between the *Bacillus* spp. groups (*p* > 0.05). Kanamycin resistance was significantly greater in the other *Bacillus* group compared to the *B*. *cereus* and *B*. *subtilis* groups (p < 0.05).

### Abundance of Antibiotic resistant *Bacillus* isolates from different meat sources

*Bacillus* spp. antimicrobial susceptibility results were stratified by isolate, source (raw chicken, local beef meat and imported beef meat) and *Bacillus* spp. group (Table S3).

The different source of meat showed few statistically significant variations in abundance of isolates resistant to the 10 antibiotics tested (Fig. 4). The *Bacillus* spp. isolates from raw retail chicken showed significantly fewer incidences of resistance to the antibiotic cephalothin (*p* < 0.05) compared to the isolates from local beef and imported beef. Isolated *Bacillus* spp. from local beef meat showed significantly more isolates resistant to oxacillin (*p* < 0.05) compared to isolates from raw chicken and no significant difference compared to imported beef (*p* > 0.15). The *Bacillus* spp. isolated from imported beef showed significantly fewer isolates with nalidixic acid resistance compared to isolates from raw chicken and local beef meat (*p* < 0.01). Isolates from local beef meat showed significantly more isolates with kanamycin resistance compared to isolates from raw chicken and imported beef meat (*p* < 0.02). There were significantly more isolates (*p* = 0.04) from imported beef with resistance to the drug combination sulfamethazole/trimethoprim compared to isolates from local beef, but there was no significant difference compared with raw chicken (*p* = 0.07).

### *Bacillus* spp. resistance profile

Multiple drug *resistance* (MDR) for *Bacillus* spp. was defined as a strain non-susceptible to ≥1 antibiotic in ≥3 antibiotic classes, extensive drug*-*resistance (XDR) was defined as a *Bacillus* spp. strain non-susceptible to ≥1 antibiotic in all but ≤2 antibiotic classes and pan drug resistance (PDR) for those *Bacillus* spp. resistant to representatives of the 8 classes of antibiotics tested. (The antibiotics and their classes are listed in Table S2). In general, the strains were resistant to 1–7 antibiotics represented in 1–5 classes. Overall, the least number of classes was one represented by penicillin and the highest number of classes was five. The total number of MDR isolates were 85 (68.5%) and no XDR nor PDR were detected (Table S3). The MDR profiles of the *Bacillus* spp. collected from chicken and beef (local and imported) meat samples, resistant to the ten tested antibiotics are summarized in Table S3.

### MAR_*index*_

To survey the relative predominance of resistant *Bacillus* isolates from raw chicken, raw local beef meat and imported frozen raw beef meat, MAR (multidrug antibiotic resistance) indices were calculated. For the raw chicken samples, the MAR index range ($\bar{x}$) for chicken was 0.1–0.7 (0.4), raw local beef meat 0.3–0.7 (0.5), and imported frozen raw beef meat 0.4–0.7 (0.5). The relatively high MAR indices indicate a high-risk level of food contamination with antibiotic resistant virulent *Bacillus* strains.

### Correlation analysis of measured variables: virulence, antibiotic resistance and meat source

Permutational MANOVA analysis using the isolated *Bacillus* spp. groups, isolate meat source, and the interaction term as terms within the model indicated significant difference in all three terms (*p* = 0.001, 0.019, and 0.002, respectively). Pairwise comparisons showed no significant difference between sources of isolates (*p* > 0.081) indicating little variation between meat sources. Comparisons between groups showed significant difference between all three groups (*p* ≤ 0.023) suggesting variation between isolates related to phenotypic and biochemical testing were related to the *Bacillus* group.

**Figure 5:**
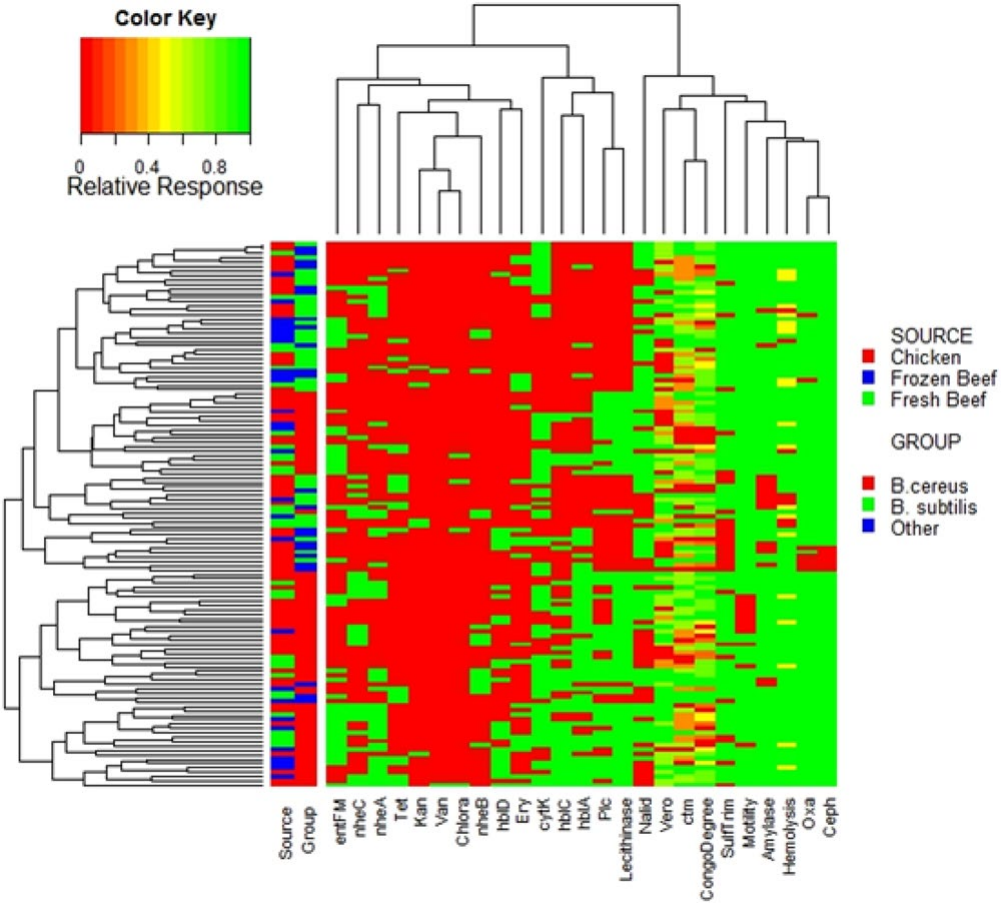
Heatmap of Individual isolates showing hierarchical clustering of isolates and factors. Binary factors (such as antibiotics or genes) indicating presence as green (relative response 1) or absence as red (relative response 0). Factors containing greater ranges were adjusted to range from 0 to 1 as indicated by color key.

**Figure 6:**
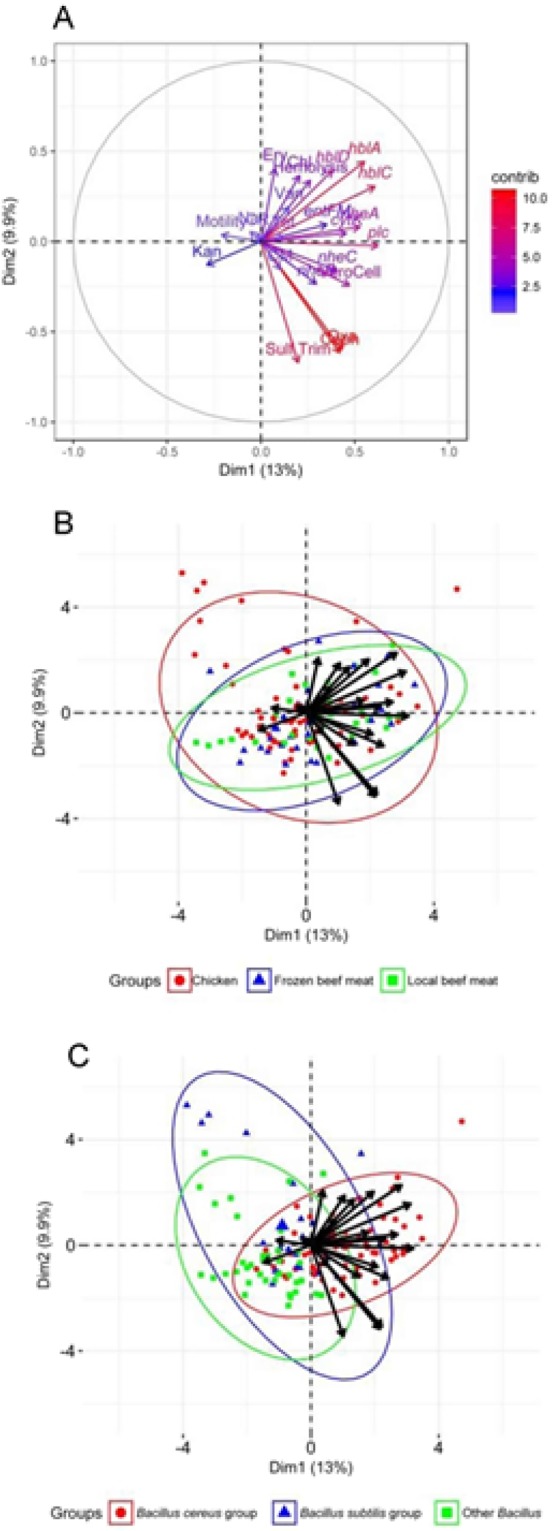
Principle component analysis of factor contribution (**A**) and relationship with groups (**B**) and meat source. (**C**) Ellipses represent 95% confidence intervals.

**Figure 7:**
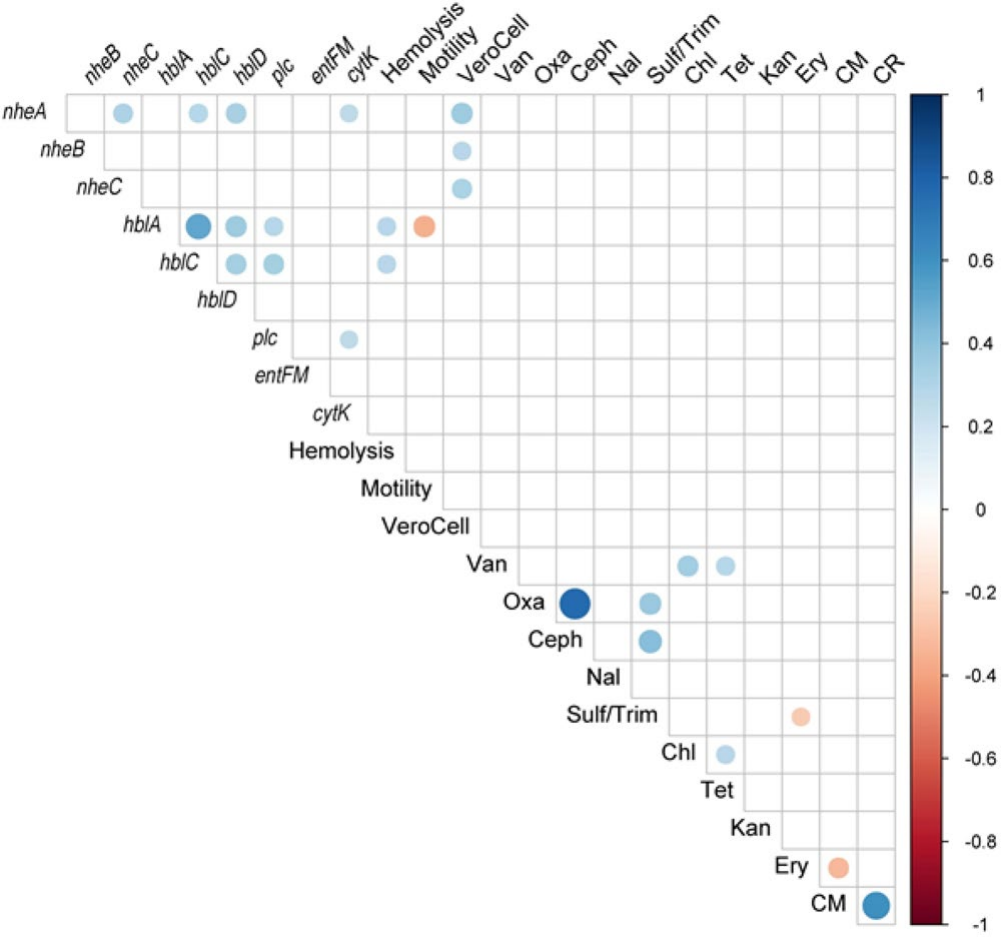
Spearman correlation matrix of phenotypic variables (antibiotic resistance, hemolytic genes, biochemical activity, and biofilm formation). Correlation matrix shows only significant (p < 0.05) correlations.

Associations of variables were identified within the hierarchical clustering of the heatmap (Fig. 5), PCA analysis (Fig. 6), and correlation matrix (Fig. 7). The correlation matrix indicated a stronger association of antibiotic resistance with one another than other variables such as hemolysis genes and biochemical tests. The lack of correlations between antibiotic resistance and other phenotypic traits or virulent genes suggest independence of antibiotic resistance to virulence.

The PCA analysis (Fig. 6) also revealed a low association of antibiotic resistance to virulence factors. The variables were also unable to separate the isolates of the *Bacillus* spp. groups into very distinct groups indicating no clear delineation of trait to a specific group. However, increased number of isolates from groups and sources may yield greater separation of the groups.

The correlation matrices (Fig. 7) indicate the *B*. *cereus* group positively correlated with the presence of the genes *hblA*, *hblC*, and *plc*, and the detection of hemolysis (*p* < 0.05), while the other *Bacillus sp*. group were negatively correlated (*p* < 0.05). Vero cell toxicity was significantly positively correlated with the presence of *nheA*, *nheB*, and *nheC* genes (*p* < 0.05) suggesting these gene products contributed to the toxicity observed. The presence of the *cytK* gene was positively correlated with the presence of *nheA* and *plc* genes (*p* < 0.05). Hemolysis was also positively correlated with the presence of the *hblA* and *hblC* genes. Additionally, the *B*. *cereus* group was positively correlated with oxacillin resistance and negatively correlated with swarming motility. The isolates of the *B*. *subtilis* group were negatively correlated with the presence of the *plc* gene and cephalothin resistance (*p* < 0.05). The correlation matrix (Fig. 7) did not identify significant correlations between antibiotic resistances and other variables with the exception of a negative correlation of erythromycin resistance and the Casein-Mannitol (CM) methods for biofilm assessment. Chloramphenicol resistance positively correlated with vancomycin, tetracycline, and erythromycin resistance (*p* < 0.05). There was a strong significant positive correlation of oxacillin resistance and cephalothin resistance (*p* < 0.05) which was evident in the MDR strains (Table S2). The CM biofilm assessment method correlated significantly with CR as expected as both are associated with degrees of biofilm formation.

## Discussion

The widespread detection of *Bacillus* spp. carrying enterotoxin-enconding genes in both chicken and beef implicates the possible hazardous impact on public health of these bacteria. We show for the first time non-*B*. *cereus* strains bearing an inventory of enterotoxigenic genes similar to *B*. *cereus* strains that could be the cause of diarrheal illness related to non-*B*. *cereus* strains as previously reported^13,25^ and thus should be considered as a risk for consumers. Further in-depth study of the sequences of the enteroxigenic genes distributed in the *Bacillus* genus may yield an explanation of the origin of the genes in non-*B*. *cereus* strains and the possibility of horizontal gene transfer between strains and species.

The *Bacillus cereus* sensu lato group are known to be involved in food poisoning^26^ although not always a notifiable disease in the majority of the international locations; therefore, incidence statistics is restrained even though fatal incidences were pronounced. Food poisoning caused by *Bacillus cereus* occurs year-round diffusely over a geographic area^27^. *Bacillus cereus* induced 0.7% of foodborne disease of the 31 primary pathogens within the US^28^ while in the Netherlands *B*. *cereus* was reported as a major causative agent in 5.4% of the foodborne outbreaks in 2006 and 32% of foodborne outbreaks in Norway in 2000^28^. *B*. *cereus* is reported as the fourth and second major cause of notified FoodBorne Organisms in the European Union and France, respectively^26^. Meat dishes were the second most commonly implicated foods in *B*. *cereus* outbreaks^29^ and 24% of *B*. *cereus* outbreaks were associated with meat or poultry dishes^27,28,30^.

In addition to the potential risk of non-*B*. *cereus* strains to consumers the specific isolation of *Paenibacillus alvei* (formerly *Bacillus alvei*) from the imported frozen meat samples is of concern. *P*. *alvei* has been one of 22 *Paenibacillus* species to be recognized as responsible for transitory or authentic infections in human clinical samples^31^. Our study showed a prevalence at 25.8% of *B*. *cereus* group and 48.6% *Bacillus* spp. of 255 meat samples consistent with earlier studies reporting rates of incidences in raw beef and chicken in different countries between 23.5% and 80%^25,30,32–40^. This variation might be due to differences in the hygienic practices executed in the meat shops in different countries and even in the same country. It should also be emphasized that, a critical control point for microbial contamination is the storage of food at ambient temperature (room temperature) of about 5–6 h and improper cooking of food before consumption which favors endospore germination producing an increase in the total *Bacillus* spp. count^39,40^. *B*. *cereus* survives not only at room temperature^41^, but it is also found in heat treated meat^33^. The *B*. *cereus* spores are resistant to heat used in cooking leading to inadequate meat preparation allowing these pathogens to germinate, replicate, and potentially produce heat stable toxins^39,42^. The initial contamination of the meat might have occurred in the environment as *Bacillus* spp. are ubiquitous bacteria in soil, in intestinal tracts of animals, and in a variety of foods and ingredients^39^. Bacterial contamination of meat can also occur during processing at the slaughter house, from the water, air, soil, the workers and equipment involved or the carcass itself^43–45^.

The virulence factors responsible for enterotoxins production in *B*. *cereus* are primarily HBL, NHE and cytotoxin K^11,12^. Diarrhea can be caused by the production of one or more enterotoxin by vegetative cells in the small intestine^1^. High detection rates between 40 and 70% in *B*. *cereus* isolated from food origin of the HBL gene complex have been reported^46–49^. Similarly, high prevalence among *B*. *cereus* isolates from food and environmental sources^49–53^, as well as in some reference strains^54^ have previously been reported for the *nheABC* and *entFM* gene complexes. All isolates of the *B*. *cereus* group carried one or more enterotoxin gene of the nine investigated in this study indicating all *B*. *cereus* group isolates could cause diarrheal illness. A similar results was obtained by Smith *et al*.^36^ and Yang *et al*.^51^. Of the non-*B*. *cereus* group there was a 72% detection of one or more enterotoxin gene(s) indicating that this group may also participate in the cause of diarrheal illness^1^. One or more gene of the NHE complex (*nheABC*) was detected in 22 (81.5%) of the *B*. *cereus* isolates indicate the presence of NHE enterotoxin. A previous study recorded that, almost all isolates contained at least one gene of the NHE complex^55^. The isolates in the current study indicated the presence of at least one gene of the HBL complex (*hblDAC*) in 19 isolates (70.4%), and similarly 73% presence of HBL complex was reported in food*-*poisoning strains^50^, whereas others found HBL complex in 65.5% and 55.2% in *B*. *cereus* isolates^30,56^. In the present study, fewer isolates contained HBL complex genes compared to NHE complex genes, which is consistent with previous findings^30^. Although Ngamwongsatit *et al*.^57^ and Vyletelova and Banyko^58^ indicated that the three genes of HBL and NHE complexes form an operon, the present and previous report by Tewari *et al*.^30^ indicate that the structural organization of these genes can be different. Interestingly, 32.3% of isolates did not show the presence of any of the HBL complex genes and produced positive β-hemolysis on blood agar. The observed hemolytic activity might be produced by other toxins such as hemolysin I (Cereolysin O)^59^, hemolysin II^60^, hemolysin III^61^ or *cyt*K^62^. The enterotoxin *cyt*K gene was found in 81.5% of the *B*. *cereus* isolates which is similar with the results reported by Ngamwongsatit *et al*.^57^, whereas others detected it in smaller percent of their isolates^63,64^. On the other hand, the *entFM* gene was found in 35.5% of our *Bacillus spp*. isolates which is far different from the 100% recorded by Ngamwongsatit *et al*.^57^ and the 93% in their *B*. *cereus* tested isolates. Smith *et al*.^36^ isolated 27 *B*. *cereus* strains out of 60 poultry samples, which contained the gene(s) for at least one of the toxins (*bceT*, *nheABC*, *hblACD*), although none of the strains contained the *cytK* gene. This was also consistent with our results with the exception with the *cytK* as previously recorded.

The hazardous impact on the food production lines as a consequence of biofilm formation comes from the fact that the biofilms are dynamic structures that can release planktonic cells able to spread and colonize new areas of the production line and equipment^65–70^. The members of *Bacillaceae* family^65,66^ have the property to attach to biotic (meat surfaces) and abiotic (meat processing equipment such as conveyor belts, tables, knives) surfaces^67^, thus the removal of biofilms in the food processing environment is critical^65–70^. In the present study, 84.7% of the *Bacillus* isolates analyzed, were able to form biofilms indicating a high potential for attachment to food processing surfaces and equipment which may lead to increase spread and incidence of illness. These results agree with previous reports that foodborne *B*. *cereus* and *B*. *thuringiensis* isolates are variable (from no or weak to strong) biofilm producers^65^. The current study highlighted that the non-*B*. *cereus* strains isolated from different meat sources were also variable biofilm producers. To our knowledge, the capacity of these species to form biofilm have not been previously studied. The disruption of biofilm formation is a key control point in eliminating the spread of *Bacillus* to food and decrease illness.

Antibiotic resistance, precipitated by the overuse of antimicrobials, may rise up from a variety of mechanisms, in particular horizontal gene transfer of virulence and antibiotic resistance genes, that is frequently facilitated with the aid of biofilm formation^71–73^. Antimicrobial resistance is a growing problem around the world and is associated with increasing mortality and medical costs^73^. Determining the resistance of *B*. *cereus* to antimicrobial agents is critical for treatment during outbreaks. The isolates from beef and chicken in this study showed resistance profile highly consistent with previous reports^49,74^. While β-lactams have become ineffective, chloramphenicol, an antibiotic in use in Egyptian clinics, would still be effective against the isolates in this study. MAR indices calculated in this study for meat are similar to values of *E*. *coli* from poultry farms^75^ but are approximately double the values for *Enterococcus* isolates^76^. The high variability in MAR index and the multi-antibiotic resistance profiles of the isolates indicate a variability in effective treatment and the importance on early determination of antibiotic resistance during infection^77^. The introduction of resistant *B*. *cereus* into the raw meat likely indicates contamination inputs from livestock and poultry operations, during transport of the meat or meat handling at the distribution outputs and poor hygienic practices in meat shops and restaurants^29,30,43,44^. Because there are no criteria for MAR_*index*_ for *B*. *cereus*, it is difficult to assess human health risks due to presence of antimicrobial resistant *B*. *cereus* in the meat.

The deficit of correlation or association of virulence factors and antibiotic resistances tested within the *Bacillus* spp. isolated in this study are consistent with observations in other species examining the correlation of virulence and antibiotic resistance^78,79^. The negative correlation of biofilm formation and resistance to the macrolide erythromycin might be selected for in part due to increased resistance to antimicrobial within the biofilm^80^. The biofilm formation *in vivo* maybe sufficient for resistance to macrolides at concentrations the isolates are exposed to, but not during our *in vitro* assay where biofilm formation is unlikely. Interestingly, He *et al*.^81^ showed that some strains of *Staphylococcus epidermidis* when exposed to sub-inhibitory concentrations of erythromycin showed increased expression of the resistance gene *ermC* with decreased biofilm formation. A similar complex relationship of biofilm formation and antibiotic resistance maybe present in the isolates in this study. The variability of virulence and antibiotic resistance profiles within the isolates are usually associated with genomic plasticity^79,82^ and *Bacillus* spp. are considered to have a plastic genome^83,84^. While plasmids may contain both virulence and antibiotic resistance genes^85,86^, the majority of plasmids currently sequenced and described within *Bacillus* spp. do not^87–90^. This lack of co-location on plasmids of virulence and antibiotic resistance genes may explain the lack of association of these factors within the isolates. Increased number of isolates and tested food products is needed to fully understand the relationship between virulence and antimicrobial resistance in *Bacillus* spp. and other pathogenic bacteria and further gene expression studies could also contribute to better understand the relationship between virulence and resistance.

## Conclusion

Considering food safety issues, this provides confirmation that *B*. *cereus* and non-*B*. *cereus* must be considered important food-borne pathogens and underlines the need to improve monitoring. The present investigation highlights for the first time an initial finding that the non-*B*. *cereus* group poses a public health risk to consumers as a consequence to their carriage of the enterotoxigenic virulence genes and exhibiting phenotypic virulence characteristics (cytotoxicity and haemolytic activity, as well as MDR and biofilm formation) as the *B*. *cereus*. In addition, the importation of meat involves a degree of disease risk to the importing country through the introduction of pathogens previously uncommon in Egypt^91^. Further research is needed to better assess the risk pose to public health by non-*B*. *cereus* species.

## Materials and Methods

### Isolation and identification of *Bacillus* spp

A total of 255 retail meat samples, comprising 156 raw chicken meat and 99 beef meat (59 local and 40 imported) were purchased from different retail outlets in the local markets of Cairo and collected and transported to the laboratory following aseptic and safety precautions.

A stomacher was used to homogenize 10 g of each sample in 90 mL of buffered peptone water (BPW) for 2 min. Heat treatment of all samples at 70 °C for 15 min to was used to eliminate vegetative cells and allow the isolation of spores^92^. The pasteurized samples were immediately placed in ice to prevent spore germination. An amount of 100 μl was spread on Mannitol–Egg Yolk–Polymyxin (MYP) agar plates and incubated at 37 °C for 24-h both aerobically and anaerobically. The plates were examined and presumptive *Bacillus* spp. were confirmed based on microscopy of Gram-stained preparations and biochemical tests^93^. A number of 15–20 colonies were randomly selected and analyzed by cell morphology, Gram staining, ability to form endospores, growth in the presence of sodium chloride, anaerobic growth, catalase and oxidase activity, Voges-Proskauer test and growth at pH 5.7^92,93^. The ability to ferment carbohydrates, starch hydrolysis, use of citrate as a carbon source, lecithinase activity, and growth inhibition by lysozyme were implemented according to methods described previously^92,93^. Reference strains for the phenotypic tests were *Bacillus cereus* ATCC 11778 and *B*. *cereus* ATCC 14579.

### Assessment of antimicrobial resistance phenotypic profile

The Kirby–Bauer disk diffusion method^94^ was used to analyze the antibiotic susceptibility patterns of the *Bacillus* spp. isolates with antibiotic discs representing the following groups/mechanisms: Group I (inhibitors to cell wall synthesis): vancomycin (30 *μ*g), penicillin G (10 U), oxacillin (1 *μ*g), cephalothin (30 µg); Group II (inhibitors to nucleic acid synthesis): nalidixic acid (30 µg), sulfamethazole/trimethoprim (0.5/9.5 *μ*g); Group III (inhibitors to protein synthesis): chloramphenicol (30 *μ*g), tetracycline (30 *μ*g), kanamycin (30 µg) and erythromycin (15 *μ*g). CLSI guidelines were used to designate *Bacillus* spp. isolates as susceptible, intermediate, or resistant to an antibiotic^95^. Recent standard definitions were implemented for the phenotypic antibiotic resistance stratification of *Bacillus* isolates^96,97^.

### Detection of phenotypic virulence factors and toxin encoding genes

#### Biofilm production

Biofilm production was assessed by the Congo red agar test and Microtiter Plate method as previously described in detail^98,99^. *Staphlococcus epidermidis* ATCC 35983, a strong slime producer, was used for positive control.

#### Congo red agar test

The 124 *B*. *cereus* and non-*B*. *cereus* isolates were cultured on brain heart infusion (BHI) agar augmented with 36 μg/ml of glucose and 0.8 μg/ml of Congo red. The plates were incubated at 37 °C for 24-h. After an additional 12-h at room temperature, slime production was assessed by colony color. Black colonies are strong slime producers, while red colonies lack slime production.

#### Microtitre Plate method

A 200 µl of the *Bacillus* isolates suspension equivalent to 0.8 McFarland (yielding 105 cfu/ml) in tryptone soy broth (TSB) was transferred to a 96-well polystyrene microtiter plate. The bacterial cell suspension was incubated for 18-h at 37 °C. The plates were decanted to remove well contents, washed with running tap water, allowed to dry for 30 min and stained with crystal violet (CV; 25%) for 5 min at room temperature before decantation washing and drying for 30 min. The plates were incubated for 1 min with 200 μl HCl 25% in each well. The absorbance at 570 nm of the CV stain were read for each well. Uninoculated wells subjected to the same procedures were used as negative controls.

### Swarming activity

Swarming motility was observed as previously described^100,101^. Briefly, an overnight culture (2 × 10^8^ cells/ml) was spotted (0.5 μl) onto the center of TrM plates (1% tryptone, 0.5%, NaCl, 0.25% agar). After 6–8 h incubation at 37 °C in a humidified chamber, the diameter of halos generated by growth were measured.

#### Detection of Hemolysin BL by blood agar plates

Hemolysin BL (HBL), a primary virulence factor, is a three-component enterotoxin consisting of *hblA*, *hblD* and *hblC* genes encoding a binding component B and two lytic components L1 and L2, respectively. The production of hemolysin BL enterotoxin of *B. cereus* isolates was demonstrated by discontinuous double hemolysis pattern on blood agar plates^102^. Overnight cultures of *Bacillus* isolates in BHI broth with 0.1% glucose (BHIG) were used to inoculate blood agar plates (Columbia agar +5% sheep-blood, Oxoid) by spot inoculation. The sheep blood agar plates were incubated at 24 °C and were frequently observed between 12 and 72 h. *B. cereus* ATCC 14579 and *B. cereus* INRA C15 were used as positive controls. *B. cereus* NC 1291 was used as a negative control.

#### Vero cell cytotoxicity assay

*Bacillus* spp. isolates were inoculated into 10 ml BHIG and incubated at 30 °C for 24-h at 150 rpm on a rotary shaker. Following incubation, 100 µl of culture was diluted into fresh 10 ml BHIG and re-incubated. The supernatant was harvested by centrifugation at 5000 g for 5 minutes and filtered with a 0.22 μm syringe filter (Millipore). Vero cells were maintained in Dulbecco’s modified Eagle’s medium with 10% fetal bovine serum (FBS) at 37 °C. Trypsinized Vero cells were diluted to 106 cells/ml in normal growth medium and 100 µl was placed in the wells of a Falcon 96-well flat bottom cell-culture plates. Plates were incubated until a confluent monolayer of cells was formed, approximately 24-h, at 37 °C^103^. Isolate-free supernatant was added (100 μl) to the first column of the 96-well plate and serially diluted by 2-fold across the columns of the plate. The plates were incubated for 18-h at 37 °C. Reactions were considered positive if greater than 50% of Vero cells showed detachment from the plate when examined under light microscopy.

### PCR detection of virulence genes for enterotoxins in *B*. *cereus*

#### DNA isolation

The 124 isolates of *Bacillus* spp. isolates were grown in 5 mL nutrient broth with shaking for 18 h at 30 °C and harvested at 5,000 g for 5 min. QIAamp DNA Mini Kit was used for genomic DNA extraction and purification. The concentration and purity of genomic DNA was measured using an Ultraspec 3000 spectrophotometer at the absorbance 260 and 280 nm. PCR was performed to detect eight enterotoxigenic encoding endotoxins genes (*hblA*, *hblC*, *hblD*, *nheA*, *nheB*, *nheC*, *cytK* and *entFM*) and one hemolytic gene encoding phospholipases (*plc*). A positive reference strain of *B*. *cereus* ATCC 14579 and sterile MilliQ water as a negative control was used in PCR analysis^104,105^. Table S4 provides details about the primers used.

### Conditions for PCR amplification

PCR reactions were comprised of 25 ng genomic DNA, 10 mM Tris-HCl (pH 8.3), 10 mM potassium chloride, 2.5 mM magnesium chloride, 0.8 mM dNTPs, 1 μM each primer, and 0.5 U of Taq DNA polymerase (Promega Corporation, WI, USA). Ultrapure sterile water was used as non-template DNA control and for the PCR component preparation. A PTC-100 Programmable Thermal Controller was used for PCR amplification. The optimized multiplexPCR utilized a standard 3-step cycling: 3 min at 95 °C; 35 cycles of (1) 94 °C for 30 sec, (2) 54 °C for 45 sec, and (3) 72 °C for 1.5 min; and a final extension at 72 °C for 5 min. PCR reaction were performed in triplicate. After amplification, gel electrophoresis was used to analyze PCR fragments for presence and correct size compared to positive control. PCR runs where a negative control showed amplification or positive control did not amplify were ignored and repeated.

### Statistical analyses

Numerical coding was used for antibiotic resistance phenotypic, biochemical results, and gene presence. Detection or absence of a specific gene (eg. *hblA*) was denoted as 1 and 0, respectively. Hemolysis, Vero Cell, Congo red (CR), and CM analysis results were exchanged with numerical values matching the degree of observed activity with 0 with absence followed by sequential integers (1, 2, etc.). For antibiotic resistance, antibiotic sensitive was denoted as 0 and resistance as 1. The open statistical program R was used for statistical analysis^106^. Multivariant statistical analysis were carried out using functions in the ‘vegan’ package^107^. Binomial similarity matrices were calculated for the isolate profiles using ‘vegdist’ function and used in permutational multivariate ANOVA (MANOVA) analyses using the ‘adonis’ function. Multiple pairwise comparisons were conducted using the ‘pairwise.perm.manova’ within the ‘RVAideMemoire’ R package^108^. A permutational ordianation method was used to determine variables statistical significant variables in further analysis utilizing ‘ordiR2step’ function. All variables, not related to species identification, were signification (p < 0.01). Principal component analysis (PCA) was performed and visualized with the R packages ‘FactoMineR’^109^ and ‘factoextra’^110^. The ‘cor’ function was used to calculate correlations and ‘cor.test’ function was used to determine significance between variables. The ‘corrplot’ function from the ‘corrplot’ package was used to visualize significant correlations^111^. For multiple comparisons, False Discovery Rate was used to adjust p-values^112^. The function ‘heatmap.3’ in the ‘GMD’ package was used to generate heatmap representations a^113^. Significant difference between data shown as percentages such as biochemical tests or antibiotic resistance by source or group was determined by proportional Z test.

### Availability of data and materials

All data generated or analyzed during this study are included in this published article.

## Electronic supplementary material

Supplementary Information
