# Supplementary material for: Poultry and beef meat as potential seedbeds for antimicrobial resistant enterotoxigenic Bacillus species: a materializing epidemiological and potential severe health hazard
Source: Sci Rep. 2018 Aug 2;8:11600. doi: 10.1038/s41598-018-29932-3 (PMC6072766; doi:10.1038/s41598-018-29932-3)
Supplement: Supplementary file 1 — Supplementary Information [file 41598_2018_29932_MOESM1_ESM.docx]

## Poultry and beef meat as potential seedbeds for antimicrobial resistant enterotoxigenic *Bacillus* species: a materializing epidemiological and potential severe health hazard

**Kamelia M. Osman^1^,** [**Anthony D. Kappell**](http://frontiersin.org/people/u/117207)**^2^, Ahmed Orab^1^, Khalid S. Al-Maary^3^, Ayman S. Mubarak^3^, Turki M. Dawoud^3^, Hassan A. Hemeg^4^_,_ Ihab M. I. Moussa^3^, Ashgan M. Hessain^5^, Hend M. Y. Yousef^6^*****,** [**Krassimira R. Hristova**](http://frontiersin.org/people/u/50161)**^7^**

**Supplemental Information**

Table S1. Distribution of enterotoxigenic genes in B. cereus and non-B. cereus strains isolated from various meat sources

| source | Species | n= of examined isolates | *nheA* | *nheB* | *nheC* | *hblA* | *hblC* | *hblD* | *Plc* | *entFM* | *cytK* |
| --- | --- | --- | --- | --- | --- | --- | --- | --- | --- | --- | --- |
| Chicken meat | *B. cereus* | 11 | **8**  **(72.7%)** | **2**  **(18.2%)** | **5**  **(45.5%)** | **9**  **(81.8%)** | **8**  **(72.7%)** | **4**  **(36.4%)** | **8**  **(72.7%)** | **6**  **(54.5%)** | **9**  **(81.8%)** |
|  |  |  |  |  |  |  |  |  |  |  |  |
|  | *B. mycoides* | 8 | ND | **2**  **(25%)** | **2**  **(25%)** | **8**  **(100%)** | **3**  **(37.5%)** | **2**  **(25%)** | **2**  **(25%)** | **2**  **(25%)** | **4**  **(50%)** |
|  | *B. thuringiensis* | 14 | **1**  **(7.1%)** | **2**  **(14.3%)** | **5**  **(35.7%)** | **7**  **(50%)** | **5**  **(35.7%)** | **2**  **(14.3%)** | **11**  **(78.6%** | **2**  **(14.3%)** | **5**  **(35.7%)** |
|  | *B. licheniformis* | 9 | **4**  **(44.4%)** | ND | **2**  **(22.2%)** | **5**  **(55.6%)** | ND | **2**  **(22.2%)** | ND | **2**  **(22.2%)** | **6**  **(66.7%)** |
|  | *B. pumilus* | 5 | **1**  **(20%)** | **1**  **(20%)** | ND | **2**  **(40%)** | **2**  **(40%)** | **2**  **(40%)** | ND | ND | **4**  **(80%)** |
|  | *B. brevis* | 4 | ND | ND | **2**  **(50%)** | ND | ND | ND | ND | ND | **2**  **(50%)** |
|  | *B. coagulans* | 7 | **3**  **(42.9%)** | ND | ND | ND | ND | ND | ND | ND | **5**  **(71.4%)** |
|  | *B. megaterium* | 5 | ND | ND | ND | ND | ND | ND | ND | **1**  **(20%)** | **2**  **(40%)** |
|  | *B. sphaericus* | 3 | **2**  **(66.7%)** | ND | **1**  **(33.3%)** | ND | ND | ND | ND | ND | **3**  **(100%)** |
|  | Total non-B. cereus | 55 | 11  (20%) | 5  (9.1%) | 12 (21.8%) | 22 (40%) | 10 (18.2%) | 8 (14.6%) | 13 (23.6%) | 9  (16.4%) | 31 (56.4%) |
| Local beef meat | *B. cereus* | 9 | **6**  **(66.7%)** | **2**  **(22.2%)** | **3**  **(33.3%)** | **5**  **(55.6%)** | **6**  **(66.7%)** | **2**  **(22.2%)** | **9**  **(100%)** | **6**  **(66.7%)** | **7**  **(77.8%)** |
|  |  |  |  |  |  |  |  |  |  |  |  |
|  | *B. mycoides* | 3 | **2**  **(66.7%)** | **1**  **(33.3%)** | **1**  **(33.3%)** | **3**  **(100%)** | **1**  **(33.3%)** | **2**  **(66.7%)** | **3**  **(100%)** | **2**  **(66.7%)** | **3**  **(100%)** |
|  | *B. thuringiensis* | 8 | **2**  **(25%)** | **1**  **(12.5%)** | **2**  **(25%)** | **5**  **(62.5%)** | **2**  **(25%)** | **1**  **(12.5%)** | **6**  **(75%)** | **4**  **(50%)** | **8**  **(100%)** |
|  | *B. licheniformis* | 2 | ND | ND | ND | **1**  **(50%)** | ND | ND | ND | **1**  **(50%)** | **2**  **(100%)** |
|  | *B. pumilus* | 2 | ND | ND | **1**  **(50%)** | **1**  **(50%)** | ND | ND | ND | **2**  **(100%)** | ND |
|  | *B. coagulans* | 6 | **2**  **(33.3%)** | ND | ND | ND | ND | ND | ND | ND | ND |
|  | Total non-B. cereus | 21 | 6  (28.6%) | 2  (9.5%) | 4 (19.0%) | 10  (47.6%) | 3 (14.3%) | 3 (14.3%) | 9 (42.9%) | 9  (42.9%) | 13  (61.9%) |
| Frozen imported beef meat | *B. cereus* | 7 | **6**  **(85.7%)** | **1**  **(14.3%)** | **2**  **(28.6%)** | **4**  **(57.1%)** | **4**  **(57.1%)** | **3**  **(42.9%)** | **7**  **(100%)** | **4**  **(57.1%)** | **6**  **(85.7%)** |
|  |  |  |  |  |  |  |  |  |  |  |  |
|  | *B. mycoides* | 1 | ND | ND | **1**  **(100%)** | **1**  **(100%)** | **1**  **(100%)** | ND | **1**  **(100%)** | ND | **1**  **(100%)** |
|  | *B. thuringiensis* | 5 | **3**  **(60%)** | ND | **1**  **(20%)** | **2**  **(40%)** | **3**  **(60%)** | **3**  **(60%)** | **3**  **(60%)** | **4**  **(80%)** | **3**  **(60%)** |
|  | *B. licheniformis* | 5 | ND | **1**  **(20%)** | ND | **2**  **(40%)** | **1**  **(20%)** | ND | ND | **3**  **(60%)** | ND |
|  | *B. stearothermophilus* | 2 | **1**  **(50%)** | ND | **1**  **(50%)** | ND | ND | **1**  **(50%)** | ND | ND | **1**  **(50%)** |
|  | *B. coagulans* | 5 | ND | **2**  **(40%)** | **1**  **(20%)** | ND | ND | ND | ND | **5**  **(100%)** | ND |
|  | *B. sphaericus* | 1 | ND | ND | ND | ND | ND | ND | ND | ND | ND |
|  | *B. alvei* | 2 | **1**  **(50%)** | **1**  **(50%)** | **1**  **(50%)** | **1**  **(50%)** | **1**  **(50%)** | **1**  **(50%)** | ND | ND | **2**  **(100%)** |
|  | Total non-B. cereus | 21 | 5  (23.8%) | 4  (19.0%) | 5  (23.8%) | 6  (28.6%) | 6  (28.6%) | 5  (23.8%) | 4  (19.0%) | 12  (57.1%) | 7  (33.3%) |
|  | Total | 124 | 42  (33.9%) | 16  (12.9%) | 31  (25%) | 56  (45.2%) | 37  (29.8%) | 25  (20.2%) | 50  (40.3%) | 44  (35.5%) | 73  (58.9%) |

ND, Not Detected

Table S2. Prevalence of antimicrobial resistance in B. cereus strains isolated from chicken and beef meat samples tested by disk diffusion method

| **On the WHO’s critically**  **important antimicrobial list (2012)** | **Antibiotic disc** | | ***Bacillus* strains**  **(number of resistant isolates/number of isolates)** | | | | | | | | | | | | |  |  |
| --- | --- | --- | --- | --- | --- | --- | --- | --- | --- | --- | --- | --- | --- | --- | --- | --- | --- |
|  |  |  | *cereus* | | *thuringiensis* | *mycoides* | | *licheniformis* | | *pumilus* | *coagulans* | *megaterium* | *sphaericus* | | *brevis* | *stearothermophilus* | *alvei* |
| **Glycopeptide** | | | | | | | | | | | | | | | |  |  |
| Critically important | Vancomycin | | | 0/27 | 1/27 | | 0/12 | | 0/16 | 0/7 | 0/18 | 0/5 | 0/4 | 0/4 | | 0/2 | 0/2 |
| **Β-lactams** | | | | | | | | | | | | | | | |  |  |
| Critically important | Penicillin | | | 11/27 | 14/27 | | 8/12 | | 9/16 | 5/7 | 7/718 | 5/5 | 3/4 | 4/4 | | 2/2 | 2/2 |
| Highly important | Oxacillin | | | 1/27 | 14/27 | | 8/12 | | 7/16 | 3/7 | 6/18 | 4/5 | 3/4 | 4/4 | | 2/2 | 2/2 |
|  | Cephalothin | | | 1/27 | 14/27 | | 8/12 | | 7/16 | 3/7 | 7/18 | 4/5 | 3/4 | 3/4 | | 2/2 | 2/2 |
| **Quinolone** | | | | | | | | | | | | | | | |  |  |
|  | Nalidixic acid | | | 8/27 | 8/27 | | 6/12 | | 8/16 | 4/7 | 6/18 | 1/5 | 1/4 | 4/4 | | 0/2 | 1/2 |
| **Sulphonamides** | | | | | | | | | | | | | | | |  |  |
| Highly important | Sulfamethoxazole/  Trimethoprim | | | 1/27 | 13/27 | | 7/12 | | 5/16 | 2/7 | 5/18 | 3/5 | 3/4 | 1/4 | | 2/2 | 1/2 |
| **Phenicols** | | | | | | | | | | | | | | | |  |  |
| Highly important | | Chloramphenicol | | 0/27 | 1/27 | | 0/12 | | 1/16 | 0/7 | 0/18 | 0/5 | 0/4 | 1/4 | | 0/2 | 0/2 |
| **Tetracycline** | | | | | | | | | | | | | | | |  |  |
| Highly important | | Tetracycline | | 1/27 | 1/27 | | 0/12 | | 1/16 | 1/7 | 1/18 | 1/5 | 1/4 | 3/4 | | 0/2 | 0/2 |
| **Aminoglycoside** | | | | | | | | | | | | | | | |  |  |
|  | | Kanamycin | | 0/27 | 1/27 | | 0/12 | | 0/16 | 0/7 | 0/18 | 0/5 | 0/4 | 0/4 | | 0/2 | 0/2 |
| **Macrolide** | | | | | | | | | | | | | | | |  |  |
| Critically important | | Erythromycin | | 1/27 | 2/27 | | 1/12 | | 3/16 | 1/7 | 3/18 | 1/5 | 0/4 | 1/4 | | 0/2 | 1/2 |

Table S3. Antimicrobial resistance profile and resistance to various numbers of antibiotics of *Bacillus* spp. isolated from chicken and beef (local and imported) meat samples (VA: Vancomycin; P: Penicillin G; OX: Oxacillin; KF: Cephalotin; NA: Nalidixic; SXT: Sulfamethoxazole/Trimethoprim; C: Chloramphenicol; TE: Tetracycline; K: Kanamycin; E: Erythromycin)

| **Origin** | **Antibiotic resistance combinations** |  |  | | ***Bacillus* Groups** | | | | | |
| --- | --- | --- | --- | --- | --- | --- | --- | --- | --- | --- |
|  |  |  | ***B.* *cereus* group** | | | | **non -*B. cereus* group** | | | |
|  |  | **n= antibiotics** | **n=antibiotic classes** | **n=isolates** | | **MDR** | | **n=isolates** | **MDR** |  |
| **Chicken meat** | P | 1 | 1 | 0 | | 0 | | 3 | 0 |  |
|  | P, NA | 2 | 2 | 0 | | 0 | | 1 | 0 |  |
|  | E, P, NA | 3 | 3 | 0 | | 0 | | 1 | 1 |  |
|  | P, NA, KF | 3 | 2 | 0 | | 0 | | 1 | 0 |  |
|  | P, NA, OX | 3 | 2 | 0 | | 0 | | 1 | 0 |  |
|  | E, P, OX, KF | 4 | 2 | 1 | | 0 | | 1 | 0 |  |
|  | P, NA, OX, KF | 4 | 2 | 0 | | 0 | | 1 | 0 |  |
|  | P, OX, SXT, KF | 4 | 2 | 7 | | 0 | | 1 | 0 |  |
|  | E, P, OX, SXT, KF | 5 | 3 | 0 | | 0 | | 1 | 1 |  |
|  | E, P, NA, OX, KF | 5 | 3 | 0 | | 0 | | 2 | 2 |  |
|  | P, OX, TE, SXT, KF | 5 | 3 | 1 | | 1 | | 2 | 2 |  |
|  | P, OX, K, SXT, KF | 5 | 3 | 1 | | 1 | | 0 | 0 |  |
|  | P, NA, OX, TE, KF | 5 | 3 | 0 | | 0 | | 1 | 1 |  |
|  | P, NA, OX, SXT,KF | 5 | 3 | 20 | | 20 | | 9 | 9 |  |
|  | E, P, NA, OX, SXT, KF | 6 | 4 | 2 | | 2 | | 2 | 2 |  |
|  | P, NA, OX, TE, SXT, KF | 6 | 4 | 0 | | 0 | | 3 | 3 |  |
|  | E, P, NA, OX, C, TE, KF | 7 | 5 | 0 | | 0 | | 2 | 2 |  |
|  | VA, E, P, OX, C, TE, KF | 7 | 5 | 1 | | 1 | | 0 | 0 |  |
|  | TOTAL |  |  | 33 | | 25 | | 33 | 24 |  |
| **Local beef meat** | P, OX, KF | 3 | 1 | 2 | | 0 | | 0 | 0 |  |
|  | P,NA, OX, KF | 4 | 2 | 3 | | 0 | | 0 | 0 |  |
|  | P, OX, K, KF | 4 | 2 | 0 | | 0 | | 1 | 0 |  |
|  | P, OX, SXT, KF | 4 | 2 | 1 | | 0 | | 0 | 0 |  |
|  | E, P, NA, OX,KF | 5 | 3 | 2 | | 2 | | 0 | 0 |  |
|  | P, NA, OX, K, KF | 5 | 3 | 0 | | 0 | | 1 | 1 |  |
|  | E, P, OX, SXT, KF | 5 | 3 | 2 | | 2 | | 0 | 0 |  |
|  | P, NA, OX, SXT, KF | 5 | 3 | 6 | | 6 | | 4 | 4 |  |
|  | P, OX, TE, SXT, KF | 5 | 3 | 1 | | 1 | | 1 | 1 |  |
|  | E, P, OX, TE, SXT, KF | 6 | 4 | 0 | | 0 | | 1 | 1 |  |
|  | E, P, NA, OX, SXT, KF | 6 | 4 | 1 | | 1 | | 0 | 0 |  |
|  | P, NA, OX, C, SXT, KF | 6 | 4 | 1 | | 1 | | 0 | 0 |  |
|  | P, NA, OX, K, SXT, KF | 6 | 4 | 0 | | 0 | | 1 | 1 |  |
|  | P, NA, OX,TE, SXT, KF | 6 | 4 | 1 | | 1 | | 0 | 0 |  |
|  | E, P, NA, OX, K, SXT,KF | 7 | 5 | 0 | | 0 | | 1 | 1 |  |
|  | TOTAL |  |  | 20 | | 14 | | 10 | 9 |  |
| **Frozen beef meat** | P, OX, SXT, KF | 4 | 2 | 5 | | 0 | | 7 | 0 |  |
|  | P, OX, TE, KF | 4 | 2 | 1 | | 0 | | 1 | 0 |  |
|  | E, P, NA, OX, KF | 5 | 3 | 0 | | 0 | | 1 | 1 |  |
|  | E, P, NA, SXT, KF | 5 | 4 | 0 | | 0 | | 1 | 1 |  |
|  | P, NA, OX, SXT, KF | 5 | 3 | 2 | | 2 | | 2 | 2 |  |
|  | P, OX, TE, SXT, KF | 5 | 3 | 2 | | 2 | | 0 | 0 |  |
|  | E, P, NA, OX, SXT, KF | 6 | 4 | 1 | | 1 | | 1 | 1 |  |
|  | E, P, OX, TE, SXT, KF | 6 | 4 | 1 | | 1 | | 0 | 0 |  |
|  | P, NA, OX, TE, SXT, KF | 6 | 4 | 1 | | 1 | | 1 | 1 |  |
|  | VA, P, NA, OX, TE, SXT, KF | 7 | 5 | 0 | | 0 | | 1 | 1 |  |
|  | TOTAL |  |  | 13 | | 7 | | 15 | 7 |  |

Table S4. Oligonucleotide primers sequences and size of the PCR-targeted products for *Bacillus* species Toxin Genes

| Target gene | Primer sequence (5′ – 3′) | Amplicon size (bp) | | References |
| --- | --- | --- | --- | --- |
| *entFM-F*  *entFM-R* | AAAGAAATTAATGGACAAACTCAAACTCA  GTATGTAGCTGGGCCTGTACGT | 609 | Sergeev et al. [104] | |
| *nheA–F*  *nheA–R* | TTTCTATCGGTACTTTAAGTAATGAAATTGTA  AACTGTTTAATGTACTTCAACGTTTGTAAC | 405 |  |  |
| *nheB–F*  *nheB–R* | TTATAAAGTAATGGCTCTATCAGCACT  TACTGCACCACCGATAATTGCAA | 750 |  |  |
| *nheC–F*  *nheC–R* | GTTCAGTTGTGAGCAGGAGCTT  AAACTATTTGTATCTTTCGCCATTCTAT | 620 |  |  |
| *hblA–F*  *hblA–R* | CGACGCTATTAACTATTACAACTGCTA  GTAACAGCATGTGCCCTTGCA | 265 |  |  |
| *hblC–F*  *hblC–R* | TATAACAAAGGAAAAGAAATTAACAACTCTA  CATGACTATTCTCCTTCTTTCGCTAA | 641 |  |  |
| *hblD–F*  *hblD–R* | TGCACAAGAAACGACCGCTCA  ATAATTTGCGCCCATTGTATTCCAT | 987 |  |  |
| *plc-F*  *plc-R* | CACTTGTAAAACAAGATCGAGTTGCA  TACACCTTTTAGCAATTTACCTTTCACGT | 727 |  |  |
| *CytK-F*  *CytK-R* | TGCTAGTAGTGCTGTAACTC  CGTTGTTTCCAACCCAGT | 881 | | Forghani et al. [105] |
